# Association of vitamin D with depression prevalence in U.S. adults: a cross-sectional analysis from NHANES 2021 to 2023

**DOI:** 10.3389/fnut.2025.1545443

**Published:** 2025-05-27

**Authors:** Chuan Huang, Jiaojiao Xu, Hai Qiu, Yuchuan Yue

**Affiliations:** ^1^School of Nursing, Chengdu University of Traditional Chinese Medicine, Chengdu, China; ^2^Affiliated Hospital of Sichuan Nursing Vocational College, The Third People's Hospital of Sichuan Province, Chengdu, China; ^3^People's Hospital of Deyang City, Deyang, China; ^4^The Fourth People's Hospital of Chengdu, Chengdu, China

**Keywords:** vitamin D, depression, mental health, cross-sectional study, NHANES

## Abstract

**Background:**

Vitamin D plays a wide array of physiological functions and is believed to influence various aspects of mental health. This cross-sectional study investigates the associations between serum levels of vitamin D isoforms—vitamin D2 (ergocalciferol) and D3 (cholecalciferol)—and the presence of depressive symptoms among U.S. adults.

**Methods:**

An analysis was conducted on data collected from 3,863 adults in the 2021–2023 National Health and Nutrition Examination Survey (NHANES). Serum vitamin D levels, represented by the combined concentrations of 25-hydroxyvitamin D2 and D3, were quantified. Depressive symptoms were evaluated through the Patient Health Questionnaire-9 (PHQ-9), where scores reaching 10 or above suggested their presence. Multivariable logistic regression models were utilized to investigate the links between vitamin D concentrations and depression, taking into account demographic and health-related factors.

**Results:**

Elevated levels of vitamin D in the serum were linked to reduced likelihood of exhibiting depressive symptoms. Specific findings indicated that increased levels of vitamin D3 correlated with a decrease in depressive symptoms, while elevated levels of vitamin D2 were linked to an increase in such symptoms. Even after accounting for potential confounding factors like age, gender, ethnicity, and socioeconomic status, these relationships remained evident.

**Conclusion:**

The study identifies distinct associations of different vitamin D isoforms with the presence of depressive symptoms, suggesting differing roles of vitamin D2 and D3 in mental health. These findings highlight the need for specific consideration of vitamin D isoforms in dietary recommendations and public health strategies aimed at mental health. Additional studies are required to clarify the underlying mechanisms responsible for these associations.

## 1 Introduction

Depression, a widespread mental health disorder, profoundly impacts global wellbeing, affecting hundreds of millions worldwide and placing a heavy burden on societal productivity and healthcare systems (1–3). Recent advancements in mental health research have highlighted the role of micronutrients (4), particularly vitamin D, in influencing psychological wellbeing (5). Vitamin D, traditionally recognized for its pivotal role in bone health (6), has also been implicated in a range of physiological functions that underscore its relevance to broader aspects of human health (7, 8), including mental wellbeing (9). The nutrient's receptors, ubiquitously expressed in the brain, suggest a potential influence on neurological pathways that regulate mood and cognitive functions (10).

Despite extensive studies investigating the correlations between serum vitamin D levels and depression, results have been conflicting. Some research supports a protective role for higher vitamin D levels in reducing the risk of depression (11, 12), while other studies report no significant associations (13, 14). Recent meta-analyses from 2019 to 2024 continue to show mixed results, with some indicating significant improvement in depressive symptoms with vitamin D supplementation, particularly in clinically depressed individuals (15, 16), while others show minimal effects in general populations without pre-existing depression (17). This discrepancy in findings may stem from the aggregation of data on different vitamin D isoforms—primarily vitamin D2 (ergocalciferol) and vitamin D3 (cholecalciferol)—which differ significantly in their sources, metabolic pathways, and physiological effects (18, 19), as well as methodological differences across studies such as baseline vitamin D status, supplementation dosages, and depression assessment tools.

Vitamin D2 and D3 not only originate from distinctly different sources but also exhibit divergent biological activities that could potentially influence their role in mental health. Vitamin D3, synthesized in the skin following sunlight exposure and found in certain animal-based foods, is generally considered more potent and has a more significant impact on maintaining overall vitamin D status than vitamin D2, which is primarily obtained from plant sources and fortified foods (20–22). The differential effects of these forms on mental health have not been thoroughly explored in the context of large-scale epidemiological studies, representing a critical gap in our understanding of vitamin D's role in depression. Recent pharmacokinetic research confirms that 25(OH)D2 has a significantly shorter half-life than 25(OH)D3, with this difference influenced by vitamin D binding protein (DBP) concentration and genotype (23). D3 demonstrates superior efficacy in raising and maintaining serum 25(OH)D levels (24). Importantly, D2 supplementation may actually lead to decreased 25(OH)D3 levels (25), potentially explaining why D2 is less effective at improving total vitamin D status.

Neurobiological mechanisms linking vitamin D to depression have been increasingly elucidated in recent research, providing a scientific basis for understanding the potentially divergent effects of D2 and D3 on mental health. Vitamin D receptors (VDRs) are expressed in multiple brain regions implicated in mood regulation, including the prefrontal cortex, hippocampus, cingulate cortex, thalamus, and amygdala (26). Recent studies have demonstrated that vitamin D plays critical roles in monoamine neurotransmission, particularly in regulating serotonin metabolism through modulation of tryptophan hydroxylase 2 (TPH2), the rate-limiting enzyme in brain serotonin synthesis (27). Furthermore, vitamin D demonstrates significant neuroprotective properties and mediates inflammatory responses in the brain, potentially counteracting the neuroinflammatory processes increasingly recognized in depression pathophysiology (28). The regulation of neurotrophic factors, particularly brain-derived neurotrophic factor (BDNF), may be a key mechanism through which vitamin D influences mood. Recent animal studies show that vitamin D3 supplementation significantly increases hippocampal BDNF expression and improves depression-like behaviors, effects that can be reversed by BDNF-blocking proteins, strongly suggesting BDNF signaling as a central mediator of vitamin D's antidepressant action (29, 30). Interestingly, BDNF Val66Met polymorphism has been shown to have gender-specific associations with depression risk (31), suggesting potential individualized approaches to vitamin D intervention.

It is also worth noting that vitamin D exerts its effects through the vitamin D receptor (VDR), and VDR gene polymorphisms, particularly FokI (rs10735810), can significantly alter an individual's response to vitamin D (32). This polymorphism modifies the VDR transcription initiation site, potentially affecting vitamin D signaling pathway efficiency. Recent research indicates an interaction between VDR gene polymorphisms and vitamin D deficiency that may jointly increase the risk of mental health disorders (33). This genetic factor might partially explain the inconsistent findings regarding vitamin D and depression, as failure to account for VDR genotypes could mask the true relationship.

Despite these advances in our understanding of vitamin D's role in mental health, significant knowledge gaps remain regarding the differential impacts of vitamin D2 and D3 on depression in large-scale population studies. Most previous research has focused on total vitamin D levels without distinguishing between isoforms, potentially obscuring important clinical differences. Additionally, studies have rarely controlled for the comprehensive range of demographic, socioeconomic, and health-related confounders that might influence both vitamin D status and depression risk, limiting the reliability of observed associations.

This research seeks to address this knowledge gap through the analysis of data from the National Health and Nutrition Examination Survey (NHANES) spanning the years 2021 to 2023. By focusing on the specific impacts of vitamin D2 and D3 on depression among U.S. adults, this research seeks to clarify these relationships and provide insights that could inform public health policies and vitamin D supplementation strategies. The findings from this study have potential clinical implications for optimizing vitamin D supplementation approaches in depression prevention and management, particularly regarding the selection of specific vitamin D forms. By elucidating the distinct associations between vitamin D isoforms and depression, we aim to provide evidence that could guide more targeted nutritional interventions in mental health care. Subsequent sections will detail the analytical methods employed, provide a comprehensive presentation of the results, and explore the implications of these findings for future studies and their practical use in public health and clinical settings.

## 2 Methods

### 2.1 Data source and participants

This study is based on a cross-sectional analysis of data collected from the 2021 to 2023 cycles of NHANES, which is overseen by the Centers for Disease Control and Prevention (CDC). NHANES aims to evaluate the health and nutritional conditions of the civilian, non-institutionalized U.S. population, utilizing detailed interviews, physical assessments, and laboratory examinations (34). Comprehensive details on the methodologies employed by NHANES, such as sampling strategies and data collection techniques, can be found on the official CDC website at https://www.cdc.gov/nchs/nhanes/.

In this analysis, we focused on adults 18 years and older with comprehensive records of serum vitamin D concentrations and depressive symptoms, evaluated using the Patient Health Questionnaire-9 (PHQ-9). Participants lacking data on these key variables or other essential covariates were excluded to maintain the integrity and robustness of our findings, ensuring that our results accurately reflect the associations between vitamin D status and depression.

**Figure 1:**
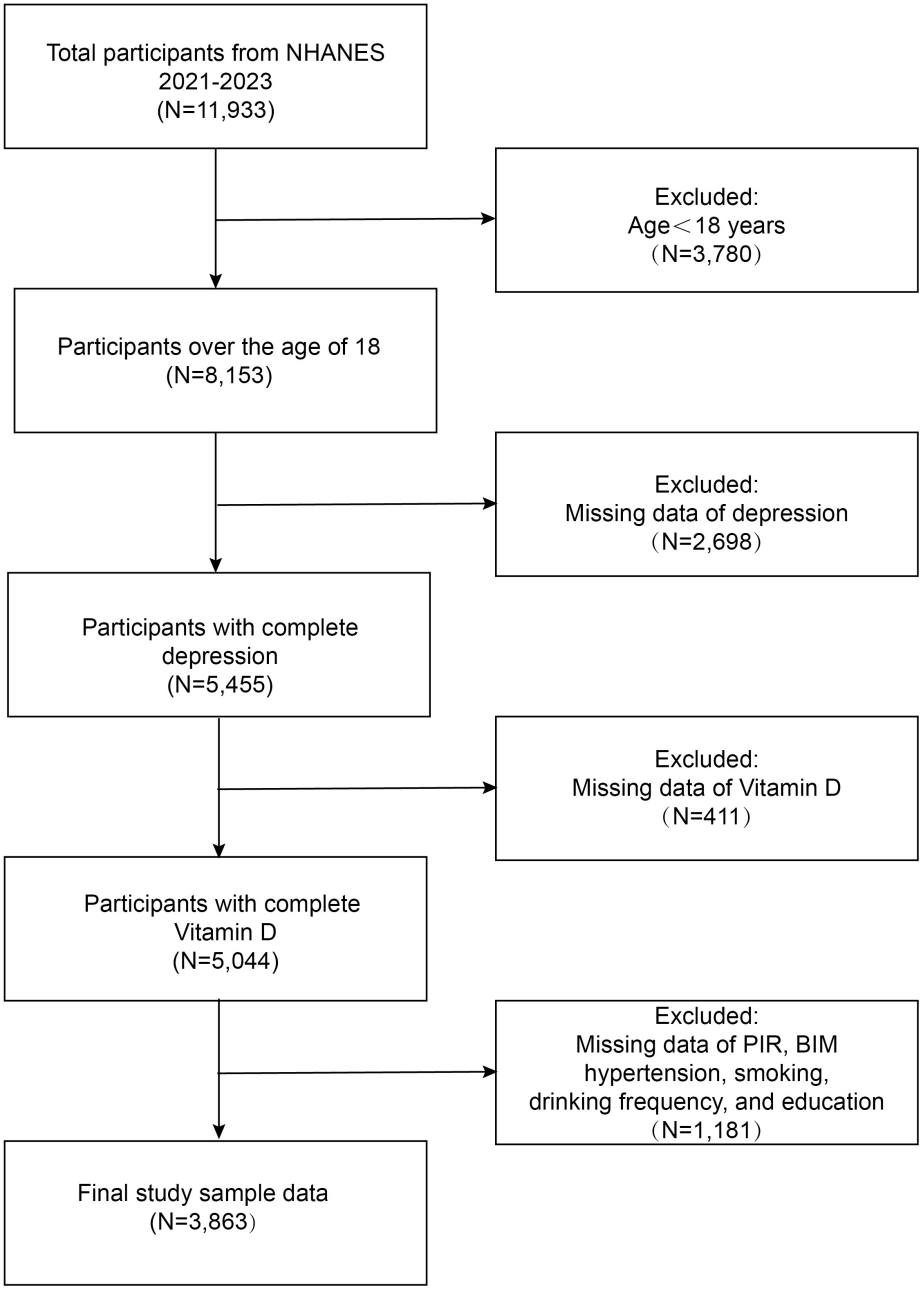
Flowchart of participants selection from NHANES 2021–2023.

The final analytic sample consisted of 3,863 participants. A detailed flowchart illustrating the inclusion and exclusion criteria for participant selection is provided in Figure 1, depicting the steps taken to form the study cohort.

### 2.2 Exposure and outcome definitions

In this research, the main exposure investigated was serum vitamin D levels, specifically analyzing the variants 25-hydroxyvitamin D3 (25OHD3) and 25-hydroxyvitamin D2 (25OHD2). These compounds were quantified via high-performance liquid chromatography-tandem mass spectrometry (HPLC-MS/MS), selected for its exceptional sensitivity and precision. This method, employed by CDC, effectively minimizes cross-reactivity between the metabolites, ensuring precise quantification. Total serum vitamin D was defined as the sum of 25OHD3 and 25OHD2 concentrations.

Depression was evaluated with the Patient Health Questionnaire-9 (PHQ-9), a widely recognized and validated instrument frequently employed in both clinical and research environments to identify depression (35). The PHQ-9 consists of nine items, each rated on a scale from 0 to 3, allowing for total scores between 0 and 27, which correspond to the DSM-5 standards for diagnosing major depressive disorder. A PHQ-9 score of 10 or above was used to classify participants as experiencing depressive symptoms, indicative of moderate to severe depression (36). Scores below 10 classified participants as non-depressed.

### 2.3 Covariates

To mitigate potential confounding in the association between serum vitamin D levels and depression, we included covariates categorized into four groups: demographic, socioeconomic, lifestyle, and health-related. Demographic factors such as age, sex, and race/ethnicity were considered to account for variations in vitamin D metabolism and depression risk across different population segments. Socioeconomic covariates included the ratio of family income to poverty (PIR) and educational attainment, which may influence health outcomes and access to resources affecting both vitamin D status and mental health.

Lifestyle factors encompassed Body Mass Index (BMI), smoking status (smoker or non-smoker), and drinking frequency (never, occasional, infrequent, or frequent), known to affect lifestyle diseases and mental health conditions. Health-related factors, specifically the presence of hypertension and diabetes, were included due to their known effects on vitamin D metabolism and their potential to exacerbate or mitigate depressive symptoms.

The selection of these covariates was driven by their documented links with depression and vitamin D metabolism, aiming to provide a thorough adjustment for factors that could influence both the exposure (vitamin D levels) and the outcome (depression). This thorough methodology provides a more precise evaluation of the actual link between vitamin D levels and depression by accounting for these factors.

### 2.4 Statistical analysis

Statistical analyses were conducted using Empower software, designed to accommodate the complex sampling design of NHANES. This approach ensures our findings are representative of the U.S. population, reflecting the stratified, multistage probability sampling structure of the survey. Three logistic regression models were developed: Model 1 (Unadjusted) provided a baseline relationship between total serum vitamin D levels and depression. Model 2 (Demographically Adjusted) included adjustments for age, sex, and race/ethnicity, key factors influencing both vitamin D metabolism and depression risk. Model 3 (Fully Adjusted) incorporated a broader set of covariates including socioeconomic status, lifestyle factors, and health-related conditions to account for potential confounding and provide accurate relationship estimates. Serum vitamin D levels were analyzed both as continuous variables for linear trends and categorically in quartiles to assess nonlinear dose-response relationships. Subgroup analyses, stratified by demographics and health-related factors, explored potential modifiers of the vitamin D-depression link. Interaction terms were tested for statistical significance to identify specific groups that might benefit differently from vitamin D regarding depression prevention.

The findings were reported as odds ratios (ORs) accompanied by 95% confidence intervals (CIs), with a significance threshold determined by a *p*-value below 0.05. Our methodology, following NHANES Analytical Guidelines, included appropriate weight adjustments to enhance the accuracy and generalizability of our results.

## 3 Results

### 3.1 Characteristics of the participants

This research involved analyzing information from a sample of 3,863 individuals, who had an average age of 53.56 years, with a standard deviation of 16.71. The cohort comprised 45.74% males and 54.26% females. Participants were categorized into two groups depending on whether they exhibited depressive symptoms, as determined by their scores on the Patient Health Questionnaire-9 (PHQ-9): 3,393 individuals without depression and 470 with depression. As shown in Table 1, significant differences were observed between these groups across several demographic and health-related variables. Notably, there were statistically significant disparities in age, sex, poverty income ratio (PIR), body mass index (BMI), education levels, smoking habits, drinking frequency, total vitamin D levels (including both D2 and D3), vitamin D3 levels specifically, and the prevalence of diabetes (all *p* < 0.05). These findings underscore distinct health and demographic profiles between participants with and without depression.

**Table 1:** Weighted characteristics of the study population based on depression.

**Characteristics**	**Non-depression**	**Depression**	***P*-value**
	***N*** = **3393**	***N*** = **470**	
Age	49.50 (48.32, 50.69)	44.37 (41.96, 46.78)	0.0003
Gender			0.0034
Male	50.56 (48.73, 52.38)	42.09 (36.08, 48.35)	
Female	49.44 (47.62, 51.27)	57.91 (51.65, 63.92)	
PIR	3.33 (3.14, 3.51)	2.44 (2.23, 2.66)	< 0.0001
BMI	29.53 (28.98, 30.08)	31.37 (30.61, 32.12)	0.0020
Race			0.7850
Mexican American	6.06 (3.24, 11.05)	7.56 (2.64, 19.80)	
Other Hispanic	7.95 (5.53, 11.31)	7.96 (5.29, 11.80)	
Non-Hispanic White	66.55 (62.20, 70.64)	63.51 (56.59, 69.92)	
Non-Hispanic Black	9.37 (7.13, 12.22)	9.43 (4.90, 17.41)	
Other Races	10.07 (8.58, 11.79)	11.54 (8.61, 15.29)	
Education			0.0089
Less than high school	6.65 (5.36, 8.22)	9.61 (6.08, 14.87)	
High school or GED	22.63 (18.80, 26.97)	27.23 (22.73, 32.25)	
Above high school	70.73 (65.41, 75.53)	63.16 (55.72, 70.02)	
Smoking			0.0475
Yes	39.08 (35.08, 43.22)	44.53 (37.30, 52.00)	
No	60.92 (56.78, 64.92)	55.47 (48.00, 62.70)	
Diabetes			0.0002
Yes	9.72 (8.25, 11.43)	14.87 (11.69, 18.73)	
No	90.28 (88.57, 91.75)	85.13 (81.27, 88.31)	
Hypertension			0.3148
Yes	30.02 (27.37, 32.82)	33.18 (27.07, 39.92)	
No	69.98 (67.18, 72.63)	66.82 (60.08, 72.93)	
Drinking frequency			0.0048
Never	13.39 (12.02, 14.89)	17.12 (12.03, 23.77)	
Occasional	20.20 (17.76, 22.89)	23.67 (19.99, 27.80)	
Infrequent	24.32 (22.47, 26.27)	27.76 (22.36, 33.90)	
Frequent	42.09 (37.99, 46.30)	31.45 (25.66, 37.87)	
Total Vitamin D (D2+D3) (ng/mL)	32.53 (31.80, 33.25)	29.15 (27.44, 30.87)	0.0009
Vitamin D2 (ng/mL)	1.53 (1.36, 1.70)	2.36 (1.54, 3.18)	0.0563
Vitamin D3 (ng/mL)	30.96 (30.24, 31.67)	26.76 (24.83, 28.70)	0.0007

Means (95% CI) were used to represent continuous variables, while proportions (95% CI) were used to represent categorical data.

### 3.2 Association of vitamin D with the likelihood of depression

The examination of the NHANES 2021-2023 dataset indicated notable correlations between levels of vitamin D, including both D2 and D3 types, and the probability of depression in different statistical models (Table 2). In Model 1, there was an observed association between increased total vitamin D levels and lower odds of depression, with each unit increase in vitamin D levels associated with a 1.9% reduction in the likelihood of depression (OR = 0.981, *P* < 0.0001). This association remained but was slightly stronger after adjusting for demographic factors in Model 2 (OR = 0.984, *P* = 0.0001) and was still evident in the comprehensive Model 3, which included adjustments for socioeconomic and health-related variables (OR = 0.991, *P* = 0.0206).

**Table 2:** The association between total vitamin D and depression.

**Exposure**	**Model 1**	**Model 2**	**Model 3**
	**OR (95CI%)**	**OR (95CI%)**	**OR (95CI%)**
	***P*** **-value**	***P*** **-value**	***P*** **-value**
**Continuous**			
Vitamin D2	1.026	1.029	1.021
	(1.014, 1.039)	(1.016, 1.042)	(1.008, 1.035)
	0.0001	< 0.0001	0.0014
Vitamin D3	0.974	0.976	0.984
	(0.966, 0.981)	(0.968, 0.984)	(0.976, 0.992)
	< 0.0001	< 0.0001	0.0001
Total vitamin D (D2+D3)	0.981	0.984	0.991
	(0.974, 0.988)	(0.977, 0.992)	(0.983, 0.999)
	< 0.0001	0.0001	0.0206
**Total vitamin D (D2**+**D3) quartile**			
Quartile 1	1.0	1.0	1.0
Quartile 2	0.904	0.934	1.056
	(0.705, 1.160)	(0.723, 1.207)	(0.810, 1.375)
	0.4289	0.6013	0.6887
Quartile 3	0.604	0.660	0.816
	(0.460, 0.793)	(0.495, 0.880)	(0.605, 1.100)
	0.0003	0.0047	0.1815
Quartile 4	0.479	0.541	0.682
	(0.360, 0.637)	(0.394, 0.741)	(0.490, 0.948)
	< 0.0001	0.0001	0.0227
*P* for trend	< 0.0001	0.0003	0.0118

Model 1 was unadjusted for variables, model 2 was adjusted for sex, age, and race, and model 3 was adjusted for the above factors smoking, drinking frequency, education, PIR, BMI, diabetes, and hypertension.

**Figure 2:**
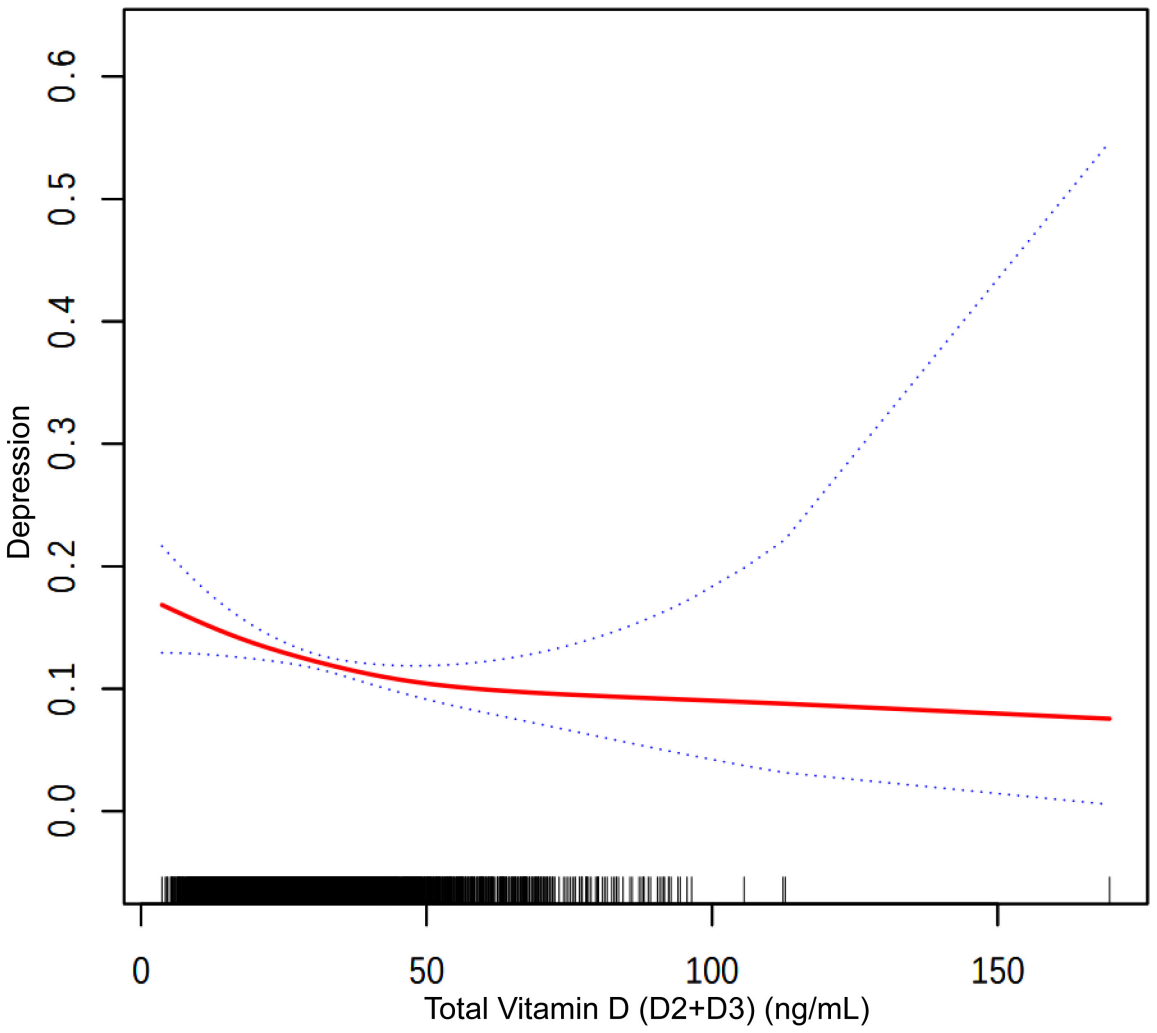
The association between total vitamin D (D2+D3) and depression. The solid red line represents the smooth curve fit between variables. Blue bands represent the 95 % of confidence interval from the fit.

Vitamin D2 levels were associated with a different pattern, where each unit increase was associated with a slight increase in the likelihood of depression across the models: 2.6% in Model 1 (OR = 1.026, *P* = 0.0001), 2.9% in Model 2 (OR = 1.029, *P* < 0.0001), and 2.1% in Model 3 (OR = 1.021, *P* = 0.0014). Figure 2 visually supports this association with a smooth curve fit illustrating an increase in depression prevalence with rising vitamin D2 levels.

**Figure 3:**
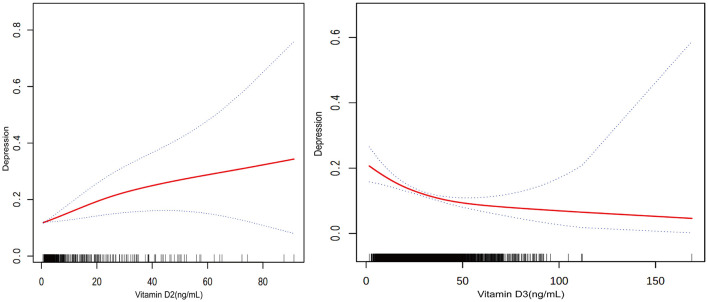
Relationship between vitamin D2 and D3 levels and the prevalence of depression. The solid red line represents the smooth curve fit between variables. Blue bands represent the 95 % of confidence interval from the fit.

In contrast, higher levels of vitamin D3 were associated with lower odds of depression in all models: 2.6% in Model 1 (OR = 0.974, *P* < 0.0001), 2.4% in Model 2 (OR = 0.976, *P* < 0.0001), and 1.6% in Model 3 (OR = 0.984, *P* = 0.0001). Figure 3 depicts this association, highlighting the relationship between higher vitamin D3 concentrations and lower depression prevalence.

Participants within the highest quartile of total vitamin D levels were observed to have a 52.1% lower prevalence of depression compared to those in the lowest quartile (OR = 0.479, *P* < 0.0001). Trend analysis across all models confirmed this relationship, with Figure 3 showing a general trend of decreasing likelihood of depression with increasing total vitamin D levels, though the curve flattens at higher levels, suggesting a leveling off of the association due to the combined effects of vitamin D2 and D3.

### 3.3 Subgroup analysis

The study explored if the relationship between vitamin D levels and the prevalence of depression differed among various demographic and health-related subgroups (Table 3). We evaluated this relationship within subgroups defined by gender, age, race, education level, poverty income ratio (PIR), body mass index (BMI), smoking habits, drinking frequency, diabetes status, and hypertension presence. The results indicated that the association between vitamin D levels and depression prevalence was consistent across all these subgroups, with no statistically significant differences observed (all *P*-values >0.05). This uniformity suggests that the relationship between vitamin D levels and depression does not significantly differ among various segments of the population.

**Table 3:** Subgroup analysis of the association between total vitamin D (D2+D3) and depression.

**Subgroup**	***N***	**OR (95%CI)**	***P*-value**	***P* for interaction**
Gender				0.3864
Male	1,767	0.983 (0.969, 0.997)	0.0162	
Female	2,096	0.990 (0.981, 0.999)	0.0348	
Age				0.5709
20–44	1,271	0.993 (0.979, 1.008)	0.3812	
45–63	1,285	0.987 (0.975, 1.000)	0.0530	
64–80	1,307	0.982 (0.968, 0.997)	0.0163	
Race				0.2334
Mexican American	228	0.960 (0.922, 1.000)	0.0499	
Other Hispanic	335	0.967 (0.932, 1.003)	0.0724	
Non-Hispanic White	2,519	0.991 (0.981, 1.001)	0.0767	
Non-Hispanic Black	389	1.002 (0.979, 1.025)	0.8756	
Other Races	392	0.981 (0.958, 1.005)	0.1150	
Education level				0.4564
Less than high school	327	1.001 (0.978, 1.025)	0.9042	
High school or GED	734	0.983 (0.967, 1.000)	0.0469	
Above high school	2,802	0.988 (0.979, 0.998)	0.0153	
Drinking frequency				0.4236
Never	645	0.984 (0.968, 1.001)	0.0576	
Occasional	805	0.983 (0.968, 0.999)	0.0316	
Infrequent	903	0.983 (0.965, 1.001)	0.0678	
Frequent	1,510	0.999 (0.984, 1.014)	0.8620	
Smoking				0.2632
Yes	1,665	0.984 (0.973, 0.995)	0.0050	
No	2,198	0.993 (0.982, 1.004)	0.1964	
Diabetes				0.3973
Yes	489	0.994 (0.978, 1.010)	0.4627	
No	3,374	0.986 (0.977, 0.995)	0.0030	
Hypertension				0.7794
Yes	1,405	0.990 (0.978, 1.001)	0.0816	
No	2,458	0.987 (0.977, 0.998)	0.0179	
PIR				0.2719
< 1.3	668	0.978 (0.963, 0.994)	0.0066	
≥1.3, < 3.5	1,435	0.989 (0.977, 1.000)	0.0545	
≥3.5	1,760	0.995 (0.981, 1.010)	0.5254	
BMI				0.9725
< 25	1,008	0.990 (0.974, 1.007)	0.2375	
≥25, < 30	1,259	0.987 (0.972, 1.003)	0.1153	
≥30	1,596	0.989 (0.978, 1.001)	0.0633	

## 4 Discussion

This research employs data from NHANES 2021-2023 to investigate the correlations between vitamin D concentrations and depression in U.S. adults. Our results highlight a complex relationship: elevated levels of vitamin D3 correlate with reduced depressive symptoms, while increased concentrations of vitamin D2 are linked to a higher prevalence of depression. These findings emphasize the intricate role of vitamin D in mental health and underscore the importance of additional research to fully understand these relationships.

We noted that elevated levels of vitamin D3 were consistently linked with reduced odds of depression in various adjusted models. This association indicates a possible connection between higher levels of vitamin D3 and a decreased risk of depression. Vitamin D3, which is primarily synthesized in the skin following sunlight exposure and found naturally in certain foods, contrasts with vitamin D2, often derived from plant sources and supplements (37–39), which showed a positive association with depression. This difference may be attributed to their distinct metabolic pathways and bioavailability (18). The divergent effects of vitamin D2 and D3 on depression may stem from subtle differences in their neurobiological mechanisms. Recent research indicates that vitamin D3 may exhibit a metabolic advantage over vitamin D2 in certain cellular contexts, potentially due to differences in CYP27B1-mediated hydroxylation and downstream signaling (40, 41). *In vitro* studies have demonstrated that 1,25(OH)_2_D3 is more potent than 1,25(OH)_2_D_2_ in activating vitamin D receptor (VDR) signaling pathways (42), which may contribute to stronger neuroprotective and anti-inflammatory effects (43, 44). Vitamin D influences the brain through several mechanisms, including regulating neurotrophic factors, influencing neuronal growth and differentiation, and modulating neurotransmitter systems such as dopamine and serotonin (45–47). Specifically, vitamin D regulates serotonin synthesis by increasing tryptophan hydroxylase 2 (TPH2) expression and may influence serotonin transporter and receptor functions (27). Animal studies demonstrate that vitamin D deficiency is associated with dysfunctional development of dopaminergic neurons, possibly through reduced expression of tyrosine hydroxylase, the rate-limiting enzyme in dopamine synthesis (48). Additionally, its role in suppressing inflammatory responses and modulating the immune system might contribute to reducing inflammation-related mood disorders by decreasing pro-inflammatory cytokines in the brain, which are implicated in the pathophysiology of depression (49–51). Furthermore, vitamin D modulates calcium signaling and calcium ion homeostasis, affecting synaptic plasticity and long-term potentiation, which are crucial for mood regulation and cognitive function (52).

A methodological consideration worth noting is our reliance on the PHQ-9 self-report questionnaire for assessing depression, which may introduce measurement bias. Although PHQ-9 is a widely validated screening tool for depression, self-reported data are susceptible to various influences, including recall bias, social desirability bias, and cultural differences. Self-reporting of depressive symptoms may vary according to individual awareness of emotional states, with certain populations potentially underreporting or being reluctant to report symptoms. Additionally, while PHQ-9 has been validated across multiple populations, its performance may differ across various cultural and socioeconomic backgrounds. We attempted to mitigate these issues by including a range of potential confounding factors and conducting subgroup analyses, but the inherent limitations of self-reported measurements should be considered when interpreting our results. Future studies should consider combining objective measurements and clinical assessments for a more comprehensive evaluation of depression status and its association with vitamin D.

The inverse relationship observed between vitamin D3 levels and depression indicates that adequate vitamin D3 levels might be considered in strategies to prevent mood disorders. This observation carries important public health implications, especially considering the common occurrence of vitamin D deficiency (53). Enhancing vitamin D status through safe sun exposure, dietary adjustments, and judicious supplementation could be strategic preventive measures against depression (54, 55). Based on our findings, we propose the following specific clinical recommendations: First, clinicians should consider routine screening of serum vitamin D levels, particularly distinguishing between 25(OH)D2 and 25(OH)D3 concentrations, for patients with depression or those at high risk. Second, when vitamin D supplementation is considered an appropriate intervention, vitamin D3 formulations should be prioritized over D2 formulations, especially for patients with existing depressive symptoms. Although optimal supplementation dosages need to be determined through further research, current evidence suggests that maintaining serum 25(OH)D levels ≥100 nmol/L may be associated with improved mental health outcomes, as higher concentrations correlate with increased probabilities of optimal psychological wellbeing (56). Personalized supplementation strategies for different populations may be necessary, considering factors such as age, gender, ethnicity, and baseline vitamin D status. Third, vitamin D3 supplementation should be integrated into comprehensive management plans for depression, as a complement to rather than a replacement for conventional treatments. Finally, it should be emphasized that for patients with severe depression, vitamin D3 supplementation should be part of a multimodal treatment approach rather than a standalone intervention. Clinical professionals should regularly monitor vitamin D levels and adjust supplementation regimens accordingly, based on individual patient characteristics and needs. Policymakers might consider promoting vitamin D3 supplementation under medical guidance as a preventive measure to enhance public mental health levels. Additionally, the observed association of high vitamin D2 levels with increased depression risk calls for cautious interpretation. This underscores the necessity for additional research to investigate how different vitamin D isoforms affect mental health. In light of these preliminary findings, healthcare professionals should be guided by the current evidence when advising on supplement choices, and public education about the varied effects of vitamin D could be beneficial (57). Recent meta-analyses support our findings, indicating that vitamin D3 supplementation effectively reduces depressive symptoms, particularly in vitamin D-deficient populations (16).

Although these findings are encouraging, it is essential to interpret the conclusions cautiously because the cross-sectional nature of our study precludes causal interpretations (58). This study design allows us to identify associations but cannot establish whether low vitamin D3 levels precede depression onset or result from behavioral changes associated with depression, such as reduced outdoor activities and altered dietary patterns. Future investigations should concentrate on longitudinal studies to evaluate whether alterations in vitamin D levels have a direct impact on depressive symptoms over time. Furthermore, exploring the biochemical pathways linking vitamin D to neurobiological changes in the brain will be crucial (59). The effectiveness and safety of vitamin D supplementation as a therapeutic intervention for depression also merit further investigation (60, 61), alongside potential gene-environment interactions that could influence individual responses to vitamin D (62–64). Randomized controlled trials specifically comparing vitamin D2 vs. D3 supplementation for depression treatment are particularly needed to confirm the differential effects observed in our study and establish optimal dosing regimens.

This study's limitations include its inability to establish causality and the potential for residual confounding, despite controlling for numerous factors. Notably, variables such as sunlight exposure, which affects vitamin D synthesis and has independent effects on mood, were not directly measured (65). Additionally, we lacked information on the duration and severity of depressive episodes, antidepressant medication use, and psychotherapy treatment, which could influence both the manifestation of depressive symptoms and potentially vitamin D metabolism. Seasonal variations in vitamin D levels and depressive symptoms, which follow similar patterns in many geographic regions, could not be fully accounted for in our cross-sectional analysis. Furthermore, while we measured serum vitamin D levels, tissue-specific concentrations and activity in the brain might be more relevant for understanding the relationship with depression. These factors highlight the necessity for careful interpretation of the results and emphasize the requirement for thorough methods in future research to bridge these gaps.

## 5 Conclusion

In conclusion, our findings from this large, representative sample of U.S. adults reveal a complex relationship between vitamin D isoforms and depression, with vitamin D3 showing protective associations and vitamin D2 demonstrating potentially adverse relationships. These contrasting effects may stem from their distinct neurobiological mechanisms, including differences in VDR signaling pathway activation potency and metabolic advantages of vitamin D3 in certain cellular contexts. While our cross-sectional design precludes causal inference, the robust associations observed across multiple adjusted models and consistency with emerging mechanistic research suggest that optimizing vitamin D3 status may be a promising adjunctive approach in depression prevention and management. Our results emphasize the importance of distinguishing between vitamin D forms in both research and clinical practice, with potential implications for screening and supplementation strategies. Future longitudinal and intervention studies, particularly randomized controlled trials comparing vitamin D2 vs. D3 supplementation, are needed to establish causality, determine optimal clinical protocols, explore biochemical pathways linking vitamin D to neurobiological changes, and investigate gene-environment interactions that may influence individual responses to vitamin D in the context of mental health.

## Data Availability

Publicly available datasets were analyzed in this study. This data can be found here: www.cdc.gov/nchs/nhanes.
